# Nutritional Profiles, Phytochemical Analysis, Antioxidant Activity and DNA Damage Protection of Makapuno Derived from Thai Aromatic Coconut

**DOI:** 10.3390/foods11233912

**Published:** 2022-12-04

**Authors:** Wannarat Phonphoem, Chomdao Sinthuvanich, Attawan Aramrak, Suteekarn Sirichiewsakul, Siwaret Arikit, Chotika Yokthongwattana

**Affiliations:** 1 Department of Biochemistry, Faculty of Science, Kasetsart University, Bangkok 10900, Thailand; 2 Department of Agronomy, Faculty of Agriculture at Kamphaeng Saen, Kasetsart University Kamphaeng Saen Campus, Nakhon Pathom 73140, Thailand

**Keywords:** antioxidant, coconut, Makapuno, nutritional value, phytochemicals

## Abstract

Makapuno is a natural mutant coconut cultivar with jelly-like endosperm. Here, we investigated the nutritional compositions, active ingredients, and antioxidant activities of Makapuno meat and water. The contents of macronutrients, sugars, vitamins, amino acids, and fatty acids were reported. We found that Makapuno meat has higher dietary fiber with lower protein and fat content compared to normal coconut meat. Medium-chain fatty acids were the major fat component of Makapuno meat and water. Phytochemical analysis revealed that while flavonoid content was lower, the total phenolic, alkaloid, and tannin contents of Makapuno meat were comparable with those of mature coconut. However, Makapuno water contained higher alkaloid content when compared to mature and young coconuts. The antioxidant activities, as examined by DPPH, FRAP, and ABTS assays, showed that Makapuno meat and water had antioxidant activities, and Makapuno water exhibited protective activity against DNA damage. Hence, this research provides the nutraceutical importance of Makapuno, which could be used in the food industry.

## 1. Introduction

Coconut (*Cocos nucifera* Linn.) is a monocot in the Arecaceae family. Coconuts are found and grown in tropical or sub-tropical regions. The coconut fruit is made up of three layers: the external layer (exocarp), the middle layer (mesocarp), and the internal layer (endocarp), which encloses the kernel or endosperm [1]. The endosperm is divided into solid and liquid coconut endosperm, known as coconut meat and coconut water, respectively. The coconut meat is usually semi-solid at a young stage and gradually thickens when the fruit reaches a mature stage as part of the coconut water is partially reduced [2].

Both coconut water and meat have high nutritional values to nourish the body and pharmaceutical properties to prevent diseases. They are rich in vitamins, fibers, phytohormones, phytochemicals, growth factors, and minerals. They also contain sugars, fatty acids (e.g., lauric acid, linoleic acid), and amino acids (e.g., lysine, arginine) [3]. Recently, the pharmaceutical properties of coconut have been reported. For example, coconut water may help protect liver cells from damage in male Sprague Dawley rats fed with high-fructose food. No damage to the liver cells was found in rats that received coconut water at 4 mL/100 g of body weight for 42 days when compared with the rats that received pure water [4]. Coconut water prevented kidney stones in rats [5] and heart disease by reducing myocardial infarction [6]. Both coconut water from young and mature fruit reduced the inflammatory effect on Sprague Dawley rats induced by intradermal injection with 0.1% acetic acid. Interestingly, the coconut water from young fruit exhibited greater anti-inflammatory activity [7]. It was also stated that coconut meat could reduce oxidative stress by the effect of L-arginine on the activity of nitric oxide synthase [8]. 

Nowadays, the elite coconut varieties with economic importance in the Southeast Asia region are the aromatic coconut and Makapuno [9,10]. Aromatic coconut is most well-known for its distinctive aroma flavor and the sweet taste of coconut water. Makapuno or curd coconut is a unique, rare coconut in which the flesh is normal in the young stage but becomes a jelly-like phenotype upon maturation (>10 months). Makapuno contains a defective function of α-D-galactosidase, an enzyme that digests galactomannan into mannan [11]. Mannan is a water-soluble carbohydrate, while galactomannan is not. Thus, Makapuno retains galactomannan, which has a jelly-like appearance in the solid endosperm. Makapuno mutation is a recessive trait, with one Makapuno fruit in every four coconut fruits. In nature, one bunch of coconut fruits will contain only one to three fruits of Makapuno. Consequently, demand has been growing for Makapuno among locals since the meat is soft, full of sweetness and aroma, and rare to find.

In the coconut industry, two stages of coconut are used, namely the young coconut and the mature coconut. Young coconut meat is consumed as fresh young meat. It is also used in Asian cooking both as a savory and sweet dish. The mature coconut meat is used for oil extraction as well as to produce coconut milk [12,13]. In Thailand, the Makapuno meat is normally consumed as a special sweet dessert, and in the Philippines as a sweet product. Additionally, the young coconut water is consumed in drinks, while mature coconut water and Makapuno water is not commonly used at all. Importantly, Makapuno has high potential for the development of food products [14] as well as for the nutraceutical and cosmeceutical industries.

The nutritional values and phytochemical components of Makapuno have been previously reported [14,15]; however, to the best of our knowledge, the information regarding antioxidant properties is scarce. Therefore, the overall objective of this study was to provide the nutritional profiles, bioactive components, and antioxidative properties of Makapuno. The results obtained from this work will shed light on the importance of Makapuno for global industrial applications, leading to a value addition for coconut farmers.

## 2. Materials and Methods

### 2.1. Materials

Gallic acid, quercetin, tannic acid, 2,2-Diphenyl-1-picrylhydrazyl, and 2,3,5-Triphenyltetrazolium chloride (TPTZ) were purchased from Sigma-Aldrich, St. Louis, MO, USA. Folin–Ciocalteu reagent was obtained from Merck KGaA, Darmstadt, Germany. Atropine was obtained from Tokyo Chemical Industry, Tokyo, Japan. 2,2-Azino-bis (3-ethylbenzothiazoline-6-sulfonic acid) diammonium salt was purchased from AppliChem GmbH, Darmstadt, Germany. Other reagents were purchased from VWR, Radnor, PA, USA.

The coconut fruits in this study were collected from three coconut trees derived from a cross between a green aromatic dwarf coconut and a tall Makapuno, which were planted in the same area. The three coconut trees were used as biological replications. Coconut fruits (young, mature, or Makapuno coconut) of each biological replicate were harvested from the same tree. The fruits were harvested in April (young coconut) or August (mature coconut) of 2022 from the KU-BEDO Coconut BioBank, Kasetsart University, Kamphaeng Saen Campus, Nakhon Pathom, Thailand (N 14.015619 E 99.958970). Young coconut fruits were harvested at 6 months old. Makapuno and mature coconuts were harvested at 10 months old. For analysis of ash, calories, carbohydrates, protein, total dietary fiber, total sugars, vitamins, and amino acids, only Makapuno samples were analyzed; seven fruits were pooled in each biological replicate. For the analysis of fatty acids, only Makapuno and mature coconut samples were analyzed; seven fruits were pooled for Makapuno and four fruits were pooled for mature coconut in each biological replicate. For the determination of phytochemical constituents, antioxidant activities, and DNA protection effects, samples were pooled from four fruits of either young, mature, or Makapuno in each biological replicate.

### 2.2. Nutritional Value Analysis

The coconut meat and water were separately collected immediately after harvesting. A total of three biological replicates were analyzed. The pooled samples were stored at −20 °C during analysis. Nutritional value analysis was carried out by the Central Laboratory (Thailand) Co., Ltd. (Bangkok, Thailand) within two weeks of sample collection. Analysis of ash, calories, carbohydrates, protein, total dietary fiber, total sugars, and fatty acids was based on AOAC (AOAC International). Vitamin B complex was determined based on [16]. Vitamin E (alpha-tocopherol) was analyzed based on [17]. Amino acid profiles were determined by an amino acid analyzer technique [18]. Tryptophan content was determined by a method based on [19]. 

### 2.3. Sample Preparations

#### 2.3.1. Preparation of Coconut Meat and Methanolic Extracts

The fresh coconut meat and the freeze-dried meat were studied. The meats were ground into powder. Methanol was added to the samples (5 mL per 1 g of meat weight). The samples were incubated overnight at room temperature, followed by centrifugation at 10,000 rpm at 4 °C for 10 min. The supernatant was collected and the pellet was used for another round of extraction. Methanol was added to the pellet (5 mL per 1 g of meat weight) and further incubated at room temperature for 3 h. The samples were centrifuged at 10,000 rpm at 4 °C for 10 min and the supernatant was collected and pooled with the supernatant from the first round.

#### 2.3.2. Preparation of Coconut Water

Coconut water was separately analyzed with 2 different preparations. The first one was used directly as fresh coconut water and the second one was freeze-dried coconut water. The powder of coconut water after freeze-drying was resuspended with distilled water, and we adjusted the concentration to 10 mg/mL.

### 2.4. Determination of Phytochemical Constituents

#### 2.4.1. Total Phenolic Content

Total phenolic compounds were determined using the Folin–Ciocalteu method as previously reported [20]. An aliquot of 20 µL extract solution was combined with 100 µL of 10% (*v*/*v*) Folin–Ciocalteu reagent in a 96-well plate and incubated at room temperature for a few minutes. After that, 80 µL of 1 M sodium carbonate was added and incubated for 20 min in the dark. The absorbance of the mixture was measured at 760 nm using an Infinite M Nano microplate reader (Tecan, Switzerland). The total phenolic contents in the extracts were calculated as gallic acid equivalent (GAE) from a calibration curve (0–200 µg/mL) and the data were expressed as µg of gallic acid equivalent per 1 g (µg GAE/g) or 1 mL of sample (µg GAE/mL).

#### 2.4.2. Flavonoid Content

Total flavonoid content was determined spectrophotometrically following a previously reported method with slight modifications [21]. The samples (50 µL) were mixed with 10 µL of 10% (*v*/*v*) aluminum chloride, 150 µL of 100% methanol, and 10 µL of 1 M potassium acetate. After 15 min incubation at room temperature, the absorbance was measured at 415 nm. Total flavonoid content was calculated as quercetin equivalent per 1 g of sample (QE) using a calibration curve (0–200 µg/mL) and expressed as µg of quercetin equivalent per 1 g (µg QE/g) or 1 mL of sample (µg QE/mL).

#### 2.4.3. Tannin Content

Analysis of tannin content was carried out as previously reported [22] with slight modifications. Briefly, 10 µL of samples were mixed with 10% Folin–Ciocalteu reagent before the addition of 120 µL of distilled water and 100 µL of 7% sodium carbonate solution. The samples were then incubated for 90 min and the absorbance was measured at 760 nm. The total tannin contents in the extracts were calculated as tannic acid equivalent from a calibration curve (0–80 µg/mL) and the data were expressed as µg of tannic acid equivalent per 1 g or 1 mL of sample. 

#### 2.4.4. Alkaloids

For the determination of alkaloids, the method was adapted from [23]. First, 0.5 mL of coconut sample was mixed with 2.5 mL of 2 M phosphate buffer, pH 4.7, followed by the addition of 2.5 mL of 0.1 mM bromocresol green solution (C_21_H_14_Br_4_O_5_S). The extraction was performed using chloroform. Then, the extract (120 μL) was added into the 96-well plate and the absorbance was measured at 417 nm. Atropine was used as a standard, 0–100 µg/mL. The data were expressed as µg of atropine equivalent per 1 g or 1 mL of sample.

### 2.5. Evaluation of Antioxidant Activity

#### 2.5.1. Determination of Antioxidant Activity Using the 2,2-Diphenyl-1-picrylhydrazyl (DPPH) Radical Scavenging Method

The free radical scavenging activity was measured by 2,2′-diphenyl-1-picrylhydrazyl (DPPH) assay according to [24]. Briefly, 25 μL of the sample was mixed with 200 μL of 150 μM DPPH. The reaction mixture was shaken well and incubated in the dark for 30 min at room temperature. Then, the absorbance was taken at 517 nm. The negative control was prepared as above using absolute methanol, and 0.1% (*w*/*v*) ascorbic acid in methanol was used as a positive control (0–80 µg/mL). The scavenging activity was estimated based on the percentage of DPPH radical scavenged as the following equation:

Scavenging effect (%) = [(A_0_ − A_s_)/A_0_] × 100

where A_0_ is the absorbance of the negative control and A_s_ is the absorbance of the sample. 

#### 2.5.2. Determination of Antioxidant Activity Using the Ferric Reducing/Antioxidant Power (FRAP) Method

The antioxidant capacity of the samples was estimated following the procedure of [25] with some modifications. The FRAP reagent was prepared by mixing 25 mL of 300 mM sodium acetate buffer solution, pH 3.6, with 2.5 mL of 10 mM TPTZ and 2.5 mL of 20 mM Iron (III) chloride. Aliquots of 10 μL of sample were mixed with 140 μL FRAP reagent. The mixture was incubated for 30 min at 37 °C in the dark. The absorbance was measured at 593 nm. The blank was prepared by substituting the same amount of sample with 10 μL of absolute methanol, and 0.1% (*w*/*v*) ascorbic acid in methanol was used as a positive control (0–120 µg/mL).

#### 2.5.3. Determination of Antioxidant Activity Using the ABTS Free Radical Scavenging Method

The antioxidant activity of the sample against ABTS was determined by the method described previously [26]. A mixture of 7.4 mM ABTS and 2.6 mM potassium persulfate solution (0.5:30 ratio) was prepared and kept in the dark at room temperature for 12 h. Then, the mixture was diluted with absolute methanol until it reached absorbance values of 1.1 ± 0.02 at 734 nm. Aliquots of 10 μL of sample were mixed with 190 μL ABTS. The mixture was incubated at room temperature in the dark for 2 h. The absorbance was measured at 734 nm, and 0.1% (*w*/*v*) ascorbic acid in methanol was used as a positive control (0–80 µg/mL).

#### 2.5.4. Analysis of DNA Damage Protection

DNA damage protection was investigated following the method described by [27]. An aliquot of 120 ng pET28a(+) plasmid was mixed with 1 μL of distilled water and 4.5 μL of 0.5 mM iron (II) sulphate. The samples (1 μL) were added into the mixture, followed by 3 μL of 15% hydrogen peroxide. The reactions were then incubated at 37 °C for 25 min and the integrity of the DNA was observed using agarose gel electrophoresis.

### 2.6. Statistical Analysis

For nutritional value analysis, all data were presented as the range of at least three biological replicates. The values were expressed per 100 g wet weight. Statistical analysis between two groups (i.e., meat vs. water or mature coconut vs. Makapuno) was assessed by a two-tailed unpaired *t*-test at the level of *p* < 0.05.

For phytochemical constituents and antioxidant activities, all data were presented as the means with standard deviation of three biological replicates. For meat samples, the data were expressed on the basis of wet weight of fresh samples (FW) or dry weight of freeze-dried samples (DW). For water samples, the data were expressed on the basis of milliliter of fresh samples (mL) or dry weight of freeze-dried samples (DW). The experimental design was a completely randomized design (CRD) with a single factor of coconut type with 3 levels (young, mature, and Makapuno). Therefore, one-way ANOVA was performed, and significant differences between the means were determined by Tukey’s multiple comparisons test at the level of *p* < 0.05. 

## 3. Results and Discussion

### 3.1. Nutritional Composition

**Figure 1:**
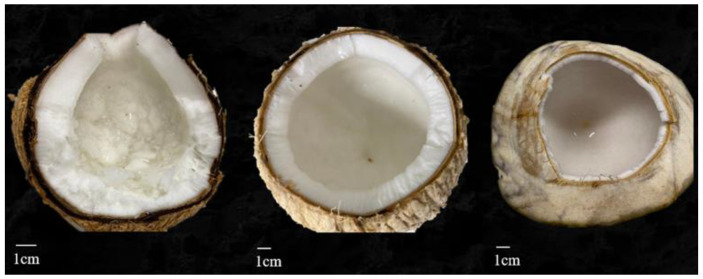
Halved fruits of 10-month-old Makapuno (**left**), 10-month-old mature coconut (**center**), and 6-month-old young coconut (**right**).

Figure 1 shows the phenotypes of Makapuno, mature coconut, and young coconut harvested from the same tree. The nutritional composition of Makapuno coconut meat and water are shown in Table 1. The values are expressed on the basis of fresh weight. Our results show that Makapuno meat contained more macronutrients than Makapuno water. Makapuno meat had slightly higher carbohydrate and dietary fiber contents with lower protein and fat compared to the reported values of mature coconut [28,29,30]. The Makapuno meat contained 74.86 ± 0.41% of moisture which was higher than that of the mature coconut (47–53%) [29]. Makapuno meat contained mainly carbohydrates (18.48 ± 2.33%) with total dietary fiber of 14.71 ± 1.98%. Sucrose was the main constituent of sugar (~71% of total sugar), followed by glucose. Fructose, maltose, and lactose were under the limit of detection. The dietary fiber of Makapuno meat, which was higher than that of mature coconut, was comparable to that of oat bran, chickpea, or other legumes [30]. The higher dietary fiber of Makapuno was most likely due to the accumulation of galactomannan, and thus has potential to be used as a functional food as well as in prebiotics to support gut health [31,32,33,34]. 

The protein content of Makapuno meat was 1.85 ± 0.17%, which was 2-fold lower than the reported value of mature coconut [29,30]. Glutamic acid was the major amino acid with an average of 337.24 ± 25.01 mg/100 g, followed by arginine and aspartic acid. This result agrees with what was reported on the amino acid profile for mature coconut [28,30]. Other detected amino acids included alanine, glycine, valine, isoleucine, leucine, histidine, and lysine. 

Vitamins B3 (nicotinic acid or niacin), B5 (pantothenic acid), B6 (pyridoxine), and C were detected in Makapuno meat. Among them, vitamin C was the main vitamin while vitamin B6 was found in a low amount. Interestingly, vitamins B1, B2, B7, B9, and E were not detected, which was different from what was reported in mature coconut [30]. 

Makapuno meat contained 3.94 ± 1.87% fat, which was about 8-fold and 3-fold lower than what has been reported for mature coconut [29,30] and other Makapuno varieties [14,15], respectively. This is reflected in the calories of Makapuno meat, which was approximately 3-fold lower than that of mature coconut [29,30]. However, the fatty acid profiles were comparable with those of the mature coconut meat (*p* < 0.05) and other Makapuno varieties (Table 2). Lauric acid (C12:0) was the dominant saturated fatty acid, and oleic acid (C18:1n9c) was the main monounsaturated fatty acid. The Makapuno and mature coconut meats contained omega-6 and omega-9 fatty acids, with no detectable omega-3. In this work, we detected a low amount of caproic acid (C6:0) in Makapuno meat and mature coconut meat, which was in accordance with [15], which reported the fatty acid profile of Makapuno in the Philippines. Approximately 80% of saturated fatty acid in Makapuno meat was medium-chain saturated fatty acids (C6–C12), which are a more rapid and direct source of energy compared to long-chain fatty acid [35]. Medium-chain fatty acid has been suggested to have potential in the treatment of metabolic disorders such as diabetes, obesity, and cardiac disease [36,37,38]. 

For Makapuno water, the main components were carbohydrates (5.57 ± 0.78% (*w*/*w*)), of which sucrose was the main constituent (~69% of total sugar). With similar content to the meat, vitamin C was dominant in the water with detectable amounts of vitamins B3, B5, and B6, while vitamins B1, B2, B7, B9, and E were not detected. Our finding indicated that both the meat and water of Makapuno have lower vitamin contents compared to common young and mature coconuts. In addition, amino acids were not detected in Makapuno water, which was in contrast with what was reported in the mature coconut. For fatty acid profiles, approximately 60% of saturated fatty acids of Makapuno were medium-chain fatty acids. Interestingly, while undetected in mature coconut water, caprylic acid (C8:0), capric acid (C10:0), and oleic acid (C18:1n9c) were detected in Makapuno water. The content of other fatty acids was comparable with mature coconut water (*p* < 0.05) [28,30].

### 3.2. Phytochemical Constituents

**Figure 2:**
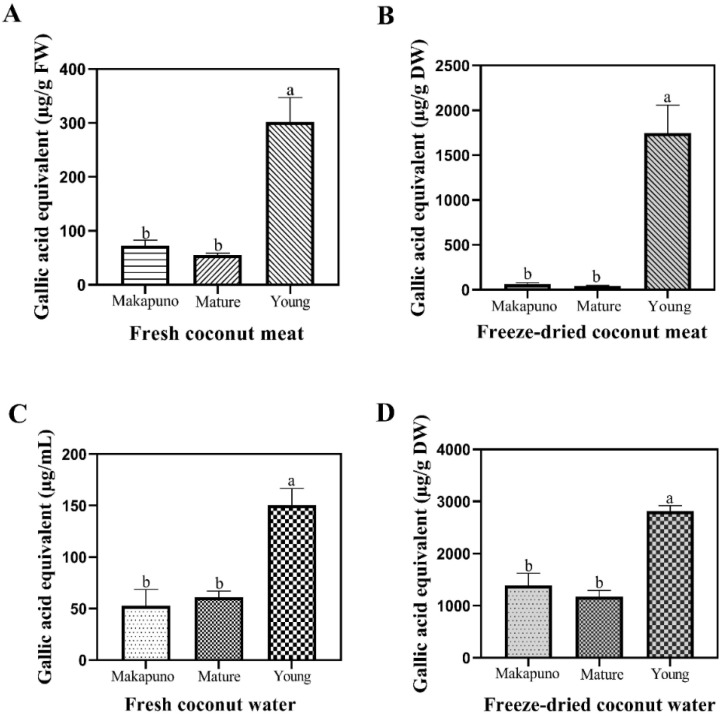
Total phenolic contents of different coconut extracts. (**A**) Fresh coconut meat sample (**B**) Freeze-dried coconut meat sample (**C**) Fresh coconut water sample (**D**) Freeze-dried coconut water sample. All experiments were performed in triplicate. Data are expressed as mean ± SE (n = 3, *p* < 0.05) for all tested. Different letters indicate mean values significantly different.

Non-communicable diseases (NCDs) have gained attention due to their high mortality rate [39]. These include cancer, diabetes, and cardiovascular disease. Dietary antioxidants are among the important factors which can effectively reduce the risk of NCDs. Fruits and vegetables are considered as good sources of natural antioxidants. Phenolic compounds are among the important phytochemicals that exhibit several biological properties, including antioxidant activities. In order to investigate the impact of food processing on phytochemical profiles, freeze-dried samples were also included in this study. Figure 2 shows that the total phenolic contents (TPC) were comparable between the Makapuno and mature coconut in both meat and water. The highest TPC was found in the meat and water of young coconuts (301.93 μg/g FW and 150.34 ± 16.23 μg/mL, respectively). This result is similar to the previous reports where a decrease in TPC was observed with the increase in maturity of coconuts [40,41]. Phenolic compounds such as quercetin, rutin, and catechin are important phytochemicals present in different plant species. Further studies are required to identify major phenolic compounds in Makapuno.

**Figure 3:**
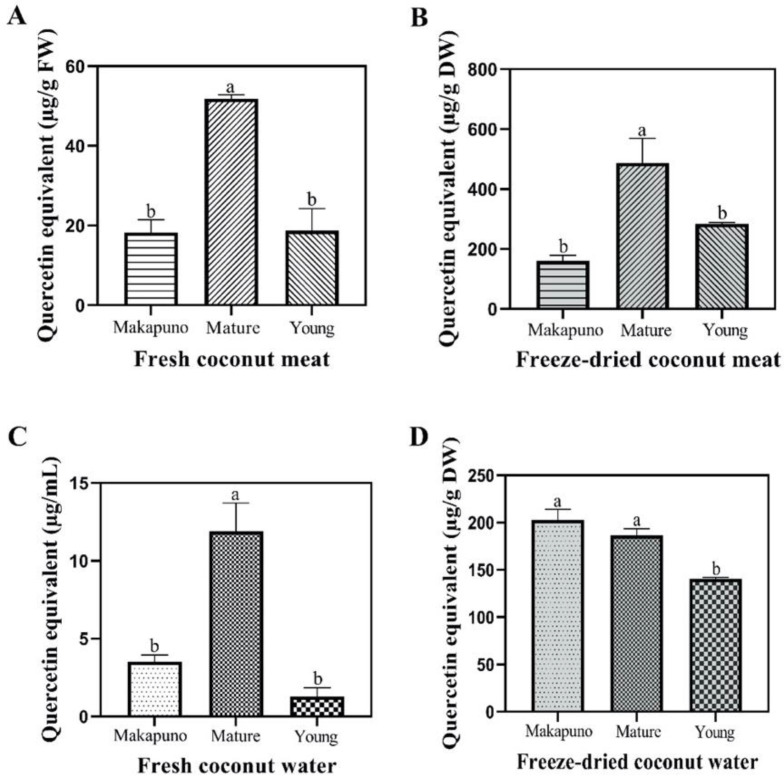
Total flavonoid contents of different coconut extracts. (**A**) Fresh coconut meat sample (**B**) Freeze-dried coconut meat sample (**C**) Fresh coconut water sample (**D**) Freeze-dried coconut water sample. All experiments were performed in triplicate. Data are expressed as mean ± SE (n = 3, *p* < 0.05) for all tested. Different letters indicate mean values significantly different.

Flavonoids are secondary metabolites in plants that attract attention due to their antioxidant activity. As shown in Figure 3, total flavonoid content (TFC) was the highest in fresh meat and water of mature coconut (51.84 ± 1.02 μg/g FW and 11.90 ± 1.81 μg/mL, respectively) compared to Makapuno and young coconuts (Figure 3A,C). For freeze-dried coconut water, however, Makapuno contained a similar amount of TFC as mature coconuts (Figure 3B,D). Differences between mature and young coconuts suggest that the maturity of coconut fruit also influences the TFC [42].

**Figure 4:**
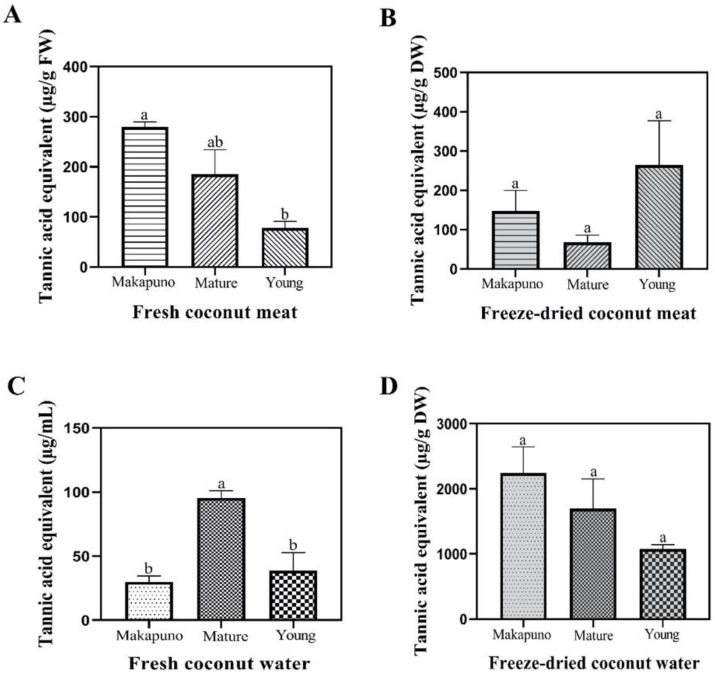
Tannin contents of different coconut extracts. (**A**) Fresh coconut meat sample (**B**) Freeze-dried coconut meat sample (**C**) Fresh coconut water sample (**D**) Freeze-dried coconut water sample. All experiments were performed in triplicate. Data are expressed as mean ± SE (n = 3, *p* < 0.05) for all tested. Different letters indicate mean values significantly different.

For the determination of tannins, the results showed that fresh Makapuno and mature coconut meat had higher total tannin content (TTC) than young coconut meat (3.58-fold and 2.37-fold, respectively) (Figure 4A). Furthermore, the highest TTC value of fresh coconut water was also found in mature coconuts (Figure 4C).

**Figure 5:**
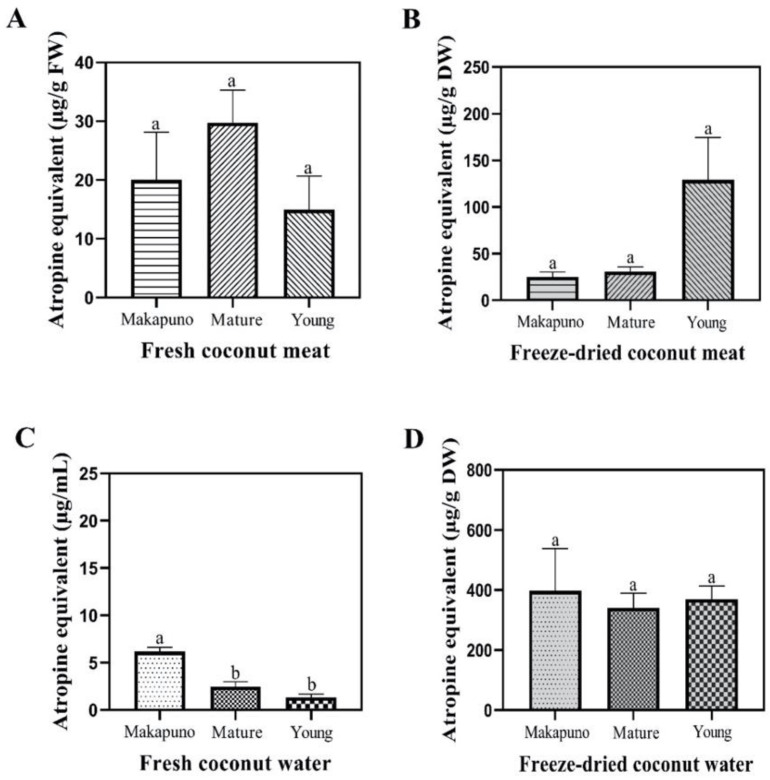
Alkaloid contents of different coconut extracts. (**A**) Fresh coconut meat sample (**B**) Freeze-dried coconut meat sample (**C**) Fresh coconut water sample (**D**) Freeze-dried coconut water sample. All experiments were performed in triplicate. Data are expressed as mean ± SE (n = 3, *p* < 0.05) for all tested. Different letters indicate mean values significantly different.

Alkaloid contents in both meat and coconut water were quantified, and no differences in alkaloid contents were found in the meat of Makapuno, mature, and young coconut (Figure 5A,B). However, Makapuno water contained a much higher amount of alkaloids (6.17 ± 0.45 μg/mL) when compared to mature and young coconut water (2.46 ± 0.53 and 1.34 ± 0.31 μg/mL, respectively) (Figure 5C).

In the present study, phenolic content, flavonoids, tannins, and alkaloids in Makapuno were analyzed spectrophotometrically to investigate the overall profiles of these phytochemicals compared to a commercially important aromatic coconut. All of these compounds were found in both meat and water of Makapuno. High tannin content was observed in the meat of Makapuno, whereas the water sample contained the highest alkaloids. These data suggested that Makapuno has potential for the management of NCDs as it contains several biological antioxidants [43]. Furthermore, the phytochemical profiles of the freeze-dried coconut were also examined for industrial applications. In the food industry, freeze-drying is a common preservation method which can be applied to many different types of food. Previous research demonstrated that a freeze-dried powdered coconut drink containing a mixture of water and meat of young coconut has similar nutritional content as the fresh coconut, with extended shelf-life [44].

### 3.3. Evaluation of Antioxidant Activities

Free radicals are harmful to cells since they adversely affect many biomolecules including DNA, lipids, and proteins. Antioxidants are small molecules as well as enzymes which play vital roles in detoxifying these toxic molecules into less harmful or even non-toxic ones. In human cells, antioxidants have protective functions against reactive oxygen species. Consuming fruits and vegetables with antioxidant activity is thought to have potential health benefits. Plants and their components contain antioxidant properties. Polyphenols, flavonoids, vitamin C, and protein extracts from plants have been demonstrated to play antioxidative roles [45,46]. The antioxidant activities of coconut have been reported, but mostly from coconut oil and coconut husk [47,48,49,50]. Only a few works reported on meat and water [51,52]. Here, we are the first to report the antioxidant activity of coconut meat and water, especially of the Makapuno variety. 

Antioxidant assays are divided into two different techniques: hydrogen atom transfer (HAT) and single electron transfer (SET) [46]. The HAT-based methods measure the capacity of an antioxidant to quench free radicals by hydrogen donation, while the SET methods detect the ability of antioxidant to transfer electrons to reduce any compound. FRAP is the SET-based assay, whereas DPPH and ABTS are assays based on mixed mechanisms [46,53]. Both coconut meat and water displayed antioxidant activities in all three varieties tested by the DPPH, FRAP, and ABTS assays. 

**Figure 6:**
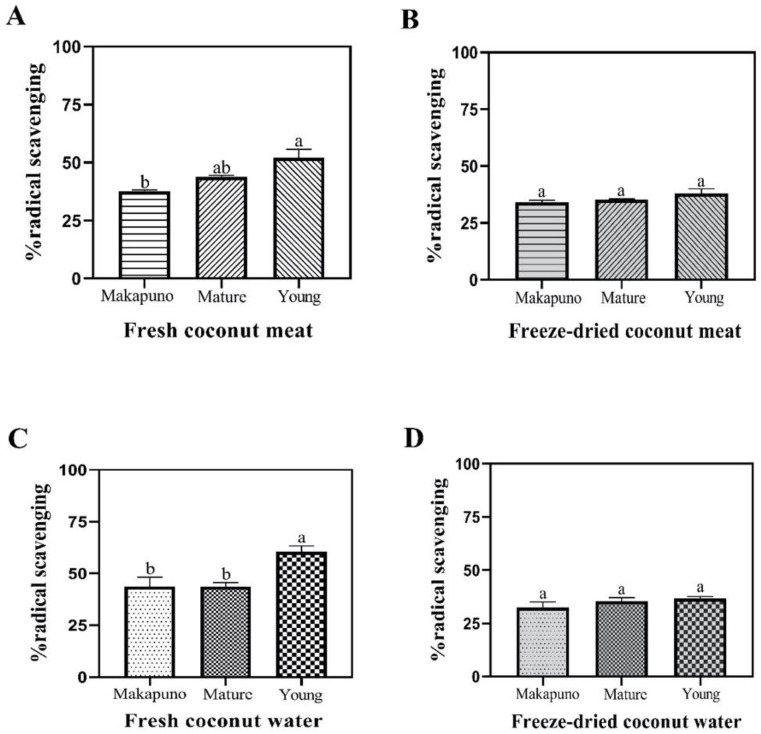
The % DPPH radical scavenging activity in coconut samples. % DPPH radical scavenging activity in coconut meats in fresh (**A**) and freeze-dried (**B**) samples. % DPPH radical scavenging activity in coconut water in fresh (**C**) and freeze-dried (**D**) samples. All experiments were performed in biological triplicate. Data are expressed as mean ± SE (n = 3, *p* < 0.05) for all tested. Different letters indicate mean values significantly different.

Assessed by DPPH radical scavenging activity, Makapuno, mature coconut, and young coconut meat as well as water showed antioxidant activity ranging from 32% to 60% radical scavenging (Figure 6). Comparing the activity among meat samples, the fresh young coconut displayed the highest DPPH radical scavenging activity (52%), followed by mature and Makapuno meat with 43% and 37% activity, respectively (Figure 6A). When the samples were freeze-dried, all of the samples displayed similar percentages of activity, with roughly 35% (Figure 6B). Comparing the coconut water, we found that the fresh young coconut water exhibited the highest DPPH radical scavenging activity (60% activity), while water from mature coconut and Makapuno displayed about 43% antioxidant activity (Figure 6C). After freeze-drying, we observed a similar trend as observed in the meat, with all of the samples displaying DPPH radical scavenging activity of 32–35% (Figure 6D).

**Figure 7:**
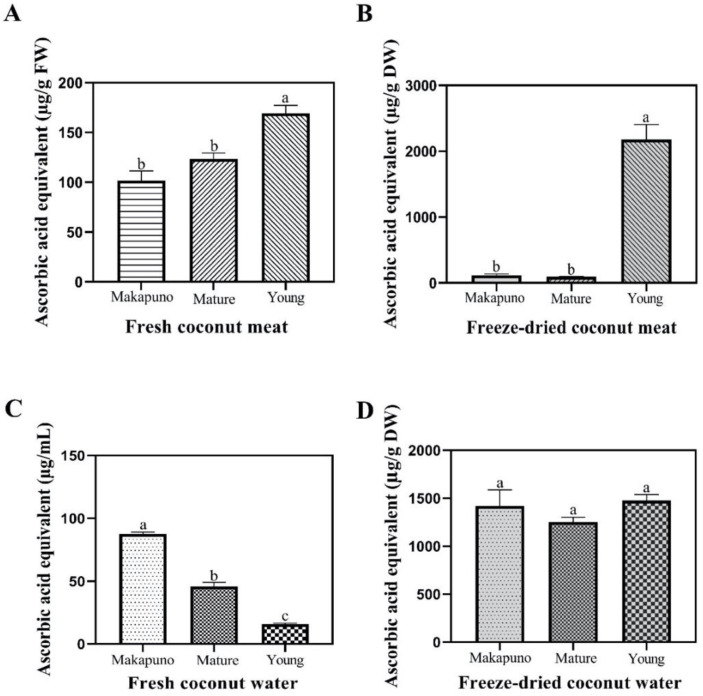
Ferric-reducing antioxidant power assay. FRAP assay of coconut meats in fresh (**A**) and freeze-dried (**B**) samples. FRAP assay of coconut water and Makapuno water in fresh (**C**) and freeze-dried (**D**) samples. All experiments were performed in triplicate. Data are expressed as mean ± SE (n = 3, *p* < 0.05) for all tested. Different letters indicate mean values significantly different.

From the FRAP assay, we observed that all of the samples from coconut meat displayed antioxidant activity. The highest activity was observed in the fresh meat of young coconut (169.17 ± 8.31 μg/g FW) (Figure 7A). After the freeze-drying process, the young coconut meat still showed the highest activity (2176.9 ± 228.32 μg/g DW) (Figure 7B). Interestingly, in the coconut water, the highest antioxidant activity was detected in the fresh Makapuno (87.75 ± 1.33 μg/mL) (Figure 7C), while all of the freeze-dried coconut waters exhibited a similar degree of antioxidant activity (Figure 7D). 

**Figure 8:**
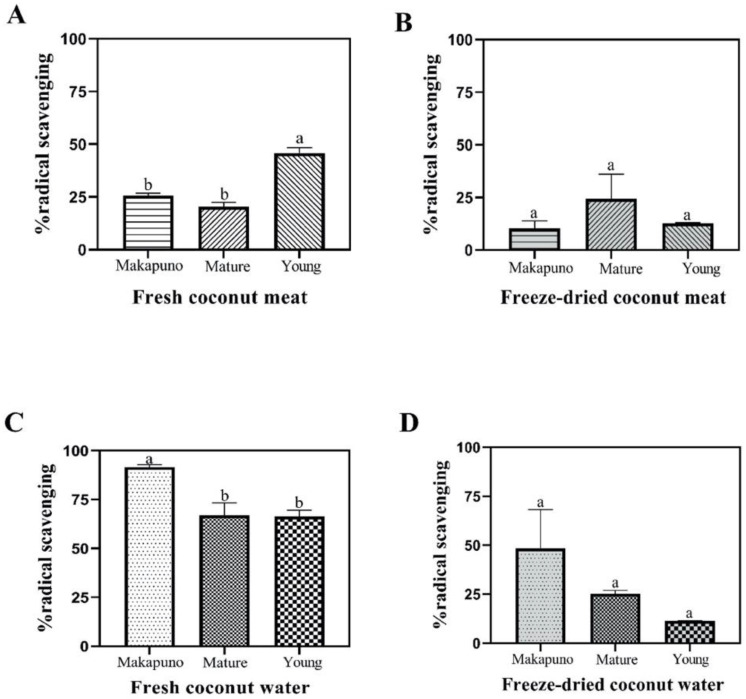
ABTS assay. ABTS assay of coconut meats in fresh (**A**) and freeze-dried (**B**) samples. ABTS assay of coconut water and Makapuno water in fresh (**C**) and freeze-dried (**D**) samples. All experiments were performed in triplicate. Data are expressed as mean ± SE (n = 3, *p* < 0.05) for all tested. Different letters indicate mean values significantly different.

Through the analysis of scavenging activity of ABTS radical in coconut samples, we observed similar trends as in the DPPH assay. Young coconut meat displayed the highest antioxidant activity (45%), followed by 25% and 20% in the extracts from Makapuno and mature coconut meat, respectively (Figure 8A). The freeze-dried meat samples showed no significant difference in the ABTS scavenging activity in all samples analyzed (Figure 8B). Regarding coconut water, the water from Makapuno had the highest antioxidant activity, displaying 91% ABTS radical scavenging, while the water from mature and young coconut exhibited about 66% scavenging activity (Figure 8C). In contrast, the freeze-dried samples of coconut water showed no statistically significant differences in antioxidant activity using ABTS assay (Figure 8D). 

**Figure 9:**
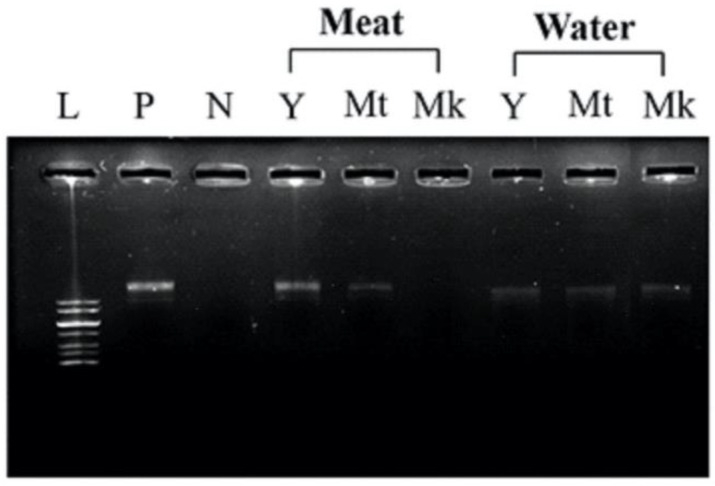
DNA damage protection assay. Lane 1: 1 kb DNA maker (1 kb); Lane 2: pET28a(+); Lane 3: pET28a(+) mixed with DI, Fe, and H_2_O_2_; Lane 4–6: pET28a(+) with coconut extract (fresh young coconut meat, fresh mature coconut meat, fresh Makapuno meat, respectively); Lane 7–9: pET28a(+) with coconut extract (fresh young coconut water, fresh mature coconut water, fresh Makapuno water, respectively).

Natural resources are considered rich sources of antioxidants, which can help prevent the DNA damage caused by free radicals. Previous study reported that a DNA duplex might form upon the binding of flavonoids with DNA, which could protect DNA from oxidative damage [54]. To further relate the antioxidant activity with the protection effect of coconut samples on the damaged biomolecules, Figure 9 shows the presence of the intact DNA band in agarose gel electrophoresis representing the protected DNA (Lane 2). We damaged DNA plasmid with the radicals generated from the Fenton reaction, as shown in Lane 3. Using this assay, we found that coconut samples exhibited DNA damage protection activity with different degrees. Methanolic extract of young coconut meat showed the highest degree of protection, as indicated by a bright DNA band, while the extract from Makapuno meat showed the least protection. Interestingly, similar protective effects were observed in water samples. These results are in agreement with the antioxidant activity observed in all samples studied. Altogether, this work showed that the coconuts, regardless of age difference, exhibit antioxidant activities with different degrees of activity. 

## 4. Conclusions

This study demonstrated that Makapuno contains a significant amount of dietary fiber and medium-chain fatty acids as well as omega-6 and omega-9 fatty acids. Phytochemical data revealed that phenolics, flavonoids, tannins, and alkaloids were found in both meat and water of Makapuno. Furthermore, Makapuno meat and water showed positive effects on protective activities against ROS scavenging and DNA damage. The assessment of nutritional and phytochemical compositions, together with antioxidant activity obtained from this study, indicates that Makapuno could be an alternative source of natural antioxidants. The knowledge from this work will pave the way to study the potential of Makapuno in preventing disease and nourishing the body, and also help stimulate the consumer market to see the benefits of this coconut variety.

## Figures and Tables

**Table 1:** Macronutrients, vitamins, and amino acid profiles of Makapuno meat and water.

Attribute	Makapuno Meat	Makapuno Water	LOD
**Macronutrient (g/100 g)**			
Ash *	0.87–0.89	0.70–0.79	-
Calories *	109.87–125.44	23.25–28.82	-
Moisture *	74.57–75.33	92.38–93.74	-
Carbohydrate *	15.80–20.07	4.70–6.22	-
Fat *	2.43–6.04	0.21–0.31	-
Protein (%N*6.25) *	1.65–1.97	0.22–0.64	-
Total Dietary Fiber *	12.43–16.00	1.22–2.09	-
**Sugar profile (g/100 g)**			
Glucose	0.81–0.88	1.04–1.33	0.30
Sucrose	2.04–2.16	2.36–2.73	0.30
Total sugar	2.89–3.02	3.43–4.06	0.30
**Vitamin profile (mg/100 g)**			
Vitamin B3 *	0.00475–0.00570	0.00709–0.00802	0.00030
Vitamin B5	0.00783–0.00910	0.00740–0.00830	0.00030
Vitamin B6	<0.00090	<0.00090	0.00030
Vitamin C	0.640–1.587	0.61100–0.82900	0.070
**Amino acid profile (mg/100 g)**			
Glutamic acid	312.36–362.38	n.d.	50.00
Arginine	<250	n.d.	100.00
Aspartic acid	<200	n.d.	100.00
Alanine	<150	n.d.	50.00
Glycine	<100	n.d.	50.00
Valine	<100	n.d.	50.00
Isoleucine	<100	n.d.	50.00
Leucine	<100	n.d.	50.00
Histidine	<100	n.d.	50.00
Lysine	<100	n.d.	50.00

A range of data from three biological replicates is presented. The samples in each biological replicate were pooled from seven fruits. Statistical differences between nutritional values of meat and water of Makapuno were assessed by a two-tailed unpaired *t*-test; * *p* value < 0.05.

**Table 2:** Fatty acid profiles in the coconut samples.

Attribute	Makapuno Meat	Mature Coconut Meat	Makapuno Water	Mature Coconut Water	LOD
**Fatty Acid Profile (g/100 g)**					
C4:0	n.d.	n.d.	n.d.	n.d.	0.01
C6:0	0.02–0.09	0.05–0.07	n.d.	n.d.	0.01
C8:0	0.25–1.22	0.65–0.84	0.01–0.02	n.d.	0.01
C10:0	0.21–0.99	0.54–0.69	0.01–0.02	n.d.	0.01
C12:0	0.83–8.35	4.39–5.96	0.08–0.13	0.04–0.05	0.01
C14:0	0.32–3.07	1.47–2.38	0.03–0.05	0.02–0.03	0.01
C16:0	0.25–1.49	0.9–1.23	0.02–0.05	0.02–0.04	0.01
C18:0	0.11–0.61	0.37–0.47	0.01–0.02	n.d.-0.01	0.01
C18:1n9c	0.15–0.98	0.55–0.86	0.01–0.03	n.d.	0.01
Saturated fat	1.83–15.88	8.56–11.66	0.15–0.25	0.08–0.13	0.01
Unsaturated fat	0.17–1.1	0.61–0.96	0.01–0.03	0.02–0.04	0.01
Monounsaturated fatty acid	0.15–0.98	0.55–0.86	0.01–0.03	0.02–0.04	0.01
Polyunsaturated fatty acid	0.02–0.12	0.01–0.06	n.d.	n.d.	0.01
Omega-6 (mg/100 g)	16.84–115.01	55.75–100.37	n.d.	n.d.	10.00
Omega-9 (mg/100 g)	153.92–984.69	549.23–856.17	14.83–29.20	16.82–39.17	10.00

A range of data from three biological replicates is presented. The Makapuno samples in each replicate were pooled from four to seven fruits. The mature coconut samples in each replicate were pooled from four fruits. Statistical differences between Makapuno and mature coconut were assessed by a two-tailed unpaired *t*-test.

## Data Availability

The date are available from the corresponding author.
